# Does Medication Sampling Improve Compliance with Brief Advice? Results from a Pragmatic Randomized Clinical Trial

**DOI:** 10.1155/2021/6638872

**Published:** 2021-03-16

**Authors:** Nathaniel J. Silvestri, Jennifer Dahne, Amy E. Wahlquist, Benjamin Toll, Matthew J. Carpenter

**Affiliations:** 1 College of Medicine, Medical University of South Carolina (MUSC), USA; 2 Department of Psychiatry and Behavioral Sciences, MUSC, USA; 3 Hollings Cancer Center, MUSC, USA; 4 Department of Public Health Sciences, MUSC, USA

## Abstract

**Introduction.:**

The 5As model is a standard component of most guidelines for tobacco treatment. Unfortunately, provider adherence to this model is modest.

**Aims.:**

Providing physicians with adjunctive tools to adhere to 5As guidelines may serve as a catalyst for brief advice delivery.

**Methods.:**

This was a secondary data analysis of a cluster randomized clinical trial assessing the uptake and impact of free nicotine replacement therapy (NRT) sampling versus standard care in primary care. Patients reported receipt of separate elements of the 5As model, assessed one month following a baseline visit. Analyses compared patients who recalled receipt of brief advice among those who received NRT vs. standard care. Additional analyses examined demographic predictors of receiving brief advice.

**Results/Findings.:**

Medication sampling did not improve compliance with ask, advise, or assess. Receipt of “assistance” was significantly higher among NRT recipients (70%) (*p* ≤ 0.0001). The NRT sampling group was more likely to have received all components (*p* = 0.004). As age increased, being asked (*p* = 0.006), advised (*p* = 0.05), and assessed (*p* = 0.003) decreased. Non-Whites reported higher rates of assessment (*p* = 0.02).

**Conclusions.:**

Provision of NRT sampling increased provider compliance with some elements of the brief advice model, thus enhancing the impact of cessation advice within primary care.

**Trial Registration.:**

This trial is registered with ClinicalTrials.gov NCT02096029.

## 1. Introduction

For the nearly 40 million smokers in the U.S., tobacco cessation remains the most important health-modifying goal [1, 2]. Efficacious cessation methods consisting of pharmacotherapies, behavioral treatments, and quitlines are all considered frontline treatment options [3]. Integrating cessation strategies within medical settings is a core recommendation of clinical practice guidelines and is often embedded within the 5As care model [4, 5]. This model recommends that providers *Ask* all patients for their smoking status, *Advise* all smokers to quit, *Assess* cessation preparedness, *Assist* in each quit attempt with referral or treatment, and *Arrange* follow-up [6]. The 5As model has substantial empirical support across multiple clinical studies [6].

Implementation of each of the 5As by providers has been varied [7]. Although they are generally proficient in determining smoking status (i.e., ask), numerous studies of both providers and patients suggest insufficient efforts are made assisting patients toward quitting [8]. For example, one study found that although tobacco use is assessed by most providers (e.g., 90%) and most provide brief advice to quit (e.g., 80%), far fewer (40%) discuss pharmacotherapy options and provide cessation follow-up [9]. While it is important for physicians to ask and advise, research suggests that these efforts are not as effective as assisting and arranging follow-up [10]. Moreover, underserved populations receive lower rates of tobacco treatment counseling [11], which may explain higher smoking rates among those populations [12].

A number of barriers limit physician adherence to the 5As. At the provider level, these include a perceived lack of time, skills, and expertise and a false belief that attempts to alter patient behavior is an unwise use of time or this task is outside of their clinical purview [13, 14]. Thus, primary care providers need explicit tools, in addition to improved education and training, to better adhere to the 5As guidelines [15].

We report herein on receipt of cessation advice from a recently completed cluster randomized clinical trial that evaluated the impact of medication sampling on patient outcomes. Both the study methods [16] and participant-based outcomes [17] are reported elsewhere. While medication sampling was hypothesized to promote smoking abstinence at the patient level (and resulted in 1.5-fold increase in cessation), it was secondarily hypothesized to increase compliance with brief advice guidelines, insofar that giving providers a concrete, immediately actionable tool to offer to their patients might facilitate a deeper and more impactful conversation about quitting. We focus specifically on the effect of medication sampling on separate elements of the 5As model. Finally, we examined individual-level predictors of receiving brief advice. These analyses are merely intended to augment existing national survey literature in this area [8], strengthened by data from a large clinical trial.

## 2. Methods

The parent trial assessed the uptake and impact of medication (NRT) sampling, delivered naturalistically and pragmatically within the primary care setting [16]. Twenty-two primary care clinics were included and randomized to either standard care (SC: receipt of the 5As and a quitline handout) or SC plus NRT sampling. During routine patient visits, enrollees were identified as smokers by each clinic’s healthcare team and were subsequently screened and consented for participation in the trial by clinic staff. Treatment delivery (SC vs. SC+NRT) was administered directly by clinic personnel within this same visit. The primary outcome of the trial was self-reported abstinence (7-day point prevalence) at the 6-month follow-up. Receipt of brief advice was assessed at the 1-month postbaseline visit.

Inclusion criteria were (1) age 18+, (2) smoker of ≥5 cigarettes per day on ≥25 days of the last 30 days, and (3) English speaking. Exclusion criteria were based on the FDA contraindications to NRT. Neither motivation to quit smoking nor a willingness to try the cessation medication was required for eligibility.

Standard of care was in concordance with the brief intervention model. Each clinic received 60–90 minutes of training on the 5As and a review of cessation medications from a tobacco treatment specialist prior to study onset. However, it was imperative to study design that healthcare providers could continue to counsel their smoking patients in their normal manner. All participants were provided with a packet that included smoking cessation information, a list of cessation medications, and contact information for the state quitline. In addition, the NRT sampling arm received a 2-week supply of both nicotine lozenge and patch (uniform 14 mg patch, 4 mg lozenge), with minimal instructions on use. Both participants and providers were aware of group assignment.

Patient response to questions regarding receipt of brief advice during their baseline visit, asked at 1-month follow-up, was recorded. Participants reported whether their provider (a) asked their smoking status, (b) advised them to quit, and (c) assessed willingness to quit, each asked via yes/no questions. Receipt of assist advice was ascertained through (d) discussion of cessation medications, (e) advice to use medications to quit smoking, or (f) provision of cessation medication. We did not formally assess follow-up (arrange) under the assumption that few patients would see their provider more than once in a 30-day window. Therefore, our analyses are restricted to 4As only and are referenced throughout as “brief advice.”

Of the 1245 participants enrolled in the study, 923 (74%) completed 1-month follow-up. We made no assumptions on missing data, and thus, analyses are based on *N* = 923. This subsample was similar to the larger study in all respects except age (responders slightly older). Receipt for each of the 4As separately and the aggregate receipt of all were compared by treatment group via generalized linear mixed models with receipt (yes/no) as the binary outcome and treatment as the main effect. All models were adjusted for the Heaviness of Smoking Index (HSI), race, and gender (in addition to including a random effect for the clinic site). To determine other factors associated with receipt of brief advice, each factor was individually examined through a generalized linear model that included a random effect for size as well as adjusted for any treatment effect that existed.

## 3. Results

The average (SD) age was 52 (13) years, with slightly more females (60%) and smoking 15 (9) cigarettes per day on average. One-third were African American (36%), 85% were insured, and 22% were unemployed.

Table 1 shows the patient-reported receipt of the 4As. The number of participants who reported they had received all 4As in aggregate from their primary care provider was significantly higher in the NRT group compared to the control group (*p* = 0.004). This in large part was due to higher rates of receiving “assistance” within the NRT sampling group (70%) vs. standard care alone (48%) (*p* ≤ 0.0001). Receipt of the other As was similar between groups.

There were several demographic factors associated with receipt of the 5As, but none were associated with receipt of all 4As (Table 2). Older patients were less likely to report that they were asked (*p* = 0.006), advised (*p* = 0.05), and assessed (*p* = 0.003). Non-Whites reported higher rates of assessment of willingness to quit (*p* = 0.02).

## 4. Discussion

This study prospectively assessed 4 of the 5As: ask, advice, assess, and assist within the context of a randomized controlled trial comparing standard of care to free NRT replacement in a primary care setting. Receipt of brief advice in aggregate was significantly improved in the NRT group compared to controls, driven largely by increased assistance. However, NRT sampling had no effect on compliance with ask, advise, or assess, despite our hypothesis that it might do so. These results add to our knowledge of tobacco control because most studies show high adherence to ask, advise, and assess and low adherence for assistance [8]. Thus, our study suggests that providing a starter package of NRT may help to increase physician provision of Assistance to smokers within a primary care setting. It is also clear that this strategy alone is unlikely to substantively improve other important elements of 5As discussion.

Curiously, only 70% of patients in the NRT group reported that they had received help with quitting, despite receiving a 2-week supply of medication. There are several possible explanations for this discrepancy. Patients may not have remembered the NRT (recall error). It is also possible that receipt of NRT from their primary care doctor was not deemed as assistance. Of note, the control group also had the opportunity to answer in the affirmative to the question of assistance as each provider had the opportunity to discuss cessation medications with their patients, even without the provision of it. In addition, while the literature deems assistance as a counseling session, medication counseling, or referral to treatment, our questionnaire was limited to a medication-focused assistance [6]. This terminology may have contributed to the lower percentage of reporting assistance among study subjects in both the control and NRT groups. Nonetheless, despite less than complete (100%) acknowledgement of receiving cessation assistance, patients who received medication samples were nearly 3 times more likely to report receipt of assistance than controls. And we postulate that if the study design had been NRT packets versus nothing, rather than cessation hand-outs, the gap would have likely been even wider.

With regard to overall predictors of 5A receipt, many of the usual disparities were confirmed: smokers of older age or with less education were less likely to receive advice to quit smoking. These associations have been documented elsewhere [11, 12] and are augmented here by the prospective randomized design (vs. population surveillance studies) and setting (primary care settings in a demographically and economically diverse southeast state) of the parent trial. Continued research is needed to explore ways to increase patient receipt of all brief advice.

In summary, this large randomized clinical trial examined medication sampling as a brief and disseminable treatment to promote smoking cessation in primary care. Patient outcomes of medication sampling were favorable [17], with an increase in medication uptake and cessation at six months. Analyses herein provide further support for medication sampling at the provider level: sampling is a useful tool that more effectively allows healthcare providers to concretely assist their patients with smoking cessation. Indeed, this study shows that providing starter packages of NRT may help to improve physician behaviors regarding tobacco treatment interventions, which may ultimately lead to larger numbers of patients quitting.

## Figures and Tables

**Table 1:** Proportion of patients in the intervention and control groups receiving brief advice^a^.

	NRT (*n* = 425)		Control (*n* = 498)		Adjusted for site+HSI, gender, race		
	*N*	%^†^	*N*	%^†^	OR	95% CI	*p* value
Asked smoking status	373	89	432	87	1.08	(0.58, 1.99)	0.8
Advised to quit	341	81	401	81	1.10	(0.61, 2.00)	0.7
Assessed motivation	317	75	351	71	1.40	(0.87, 2.27)	0.2
Assistance**	295	70	240	48	2.84	(1.69, 4.77)	<0.0001
All advice	210	50	193	39	1.72	(1.19, 2.49)	0.004

a Brief advice based on receipt of 4As (arrange not included; see text for the rationale). Each question asked at 1-month follow up to primary care (baseline) visit.

** See text for coding of assistance, across any of several specific indicators.

† Calculated from nonmissing values at 1-month visit.

**Table 2:** Factors associated with receipt of the brief advice^a^.

	Ask	Advise	Assess	Assist	All advice
Gender					
Male	Reference	Reference	Reference	Reference	Reference
Female	1.18^a^ (0.78–1.77)	0.79 (0.55–1.13)	0.98 (0.72–1.33)	**0.68 (0.51–0.91)**	0.80 (0.61–1.05)
Insurance					
Yes	Reference	Reference	Reference	Reference	Reference
No	0.67 (0.38–1.19)	0.87 (0.51–1.49)	1.25 (0.76–2.06)	1.15 (0.73–1.83)	1.09 (0.71–1.66)
Marital status					
Not married	Reference	Reference	Reference	Reference	Reference
Married/couple	1.13 (0.74–1.71)	1.24 (0.87–1.76)	0.85 (0.63–1.15)	1.00 (0.75–1.33)	1.10 (0.84–1.45)
Race					
White	Reference	Reference	Reference	Reference	Reference
Non-White	0.95 (0.59–1.52)	1.45 (0.95–2.20)	**1.51 (1.05–2.17)**	1.17 (0.83–1.64)	**1.40 (1.03–1.90)**
Education					
>HS	Reference	Reference	Reference	Reference	Reference
High school	0.88 (0.53–1.46)	1.13 (0.77–1.68)	1.10 (0.78–1.56)	1.18 (0.86–1.62)	1.32 (0.97–1.78)
<HS	**0.42 (0.25–0.71)**	0.89 (0.57–1.40)	0.76 (0.52–1.13)	1.07 (0.73–1.60)	1.00 (0.69–1.44)
Age	**0.98 (0.96–0.99)**	**0.99 (0.97–1.00)**	**0.98 (0.97–0.99)**	1.00 (0.99–1.01)	0.99 (0.98–1.00)

a Brief advice based on receipt of 4As (arrange not included; see text for the rationale). Data for all cells reflect odds ratios (95% confidence intervals). Odds ratio for each 1 yr increase in age. Note: bold odds ratios (95% CIs) denote *p* < 0.05.
